# 2390: Cardiac abnormalities drive exercise intolerance in patients with nonslcoholic fatty liver disease

**DOI:** 10.1017/cts.2017.134

**Published:** 2018-05-10

**Authors:** Justin M. Canada, Hayley Billingsly, Leo Buckley, Salvatore Carbone, Dinesh Kadariya, Benjamin Van Tassell, Antonio Abbate, Mohammad Siddiqui

## Abstract

OBJECTIVES/SPECIFIC AIMS: Nonalcoholic fatty liver disease (NAFLD) affects 1 in 3 Americans and can exist in 2 histological subtypes: simple hepatic steatosis (SHS) and nonalcoholic steatohepatitis (NASH), a clinically aggressive variant. Fatigue is the most common complaint in patients with NAFLD but the etiology of fatigue is unknown. Thus, the goal of this study was to objectively evaluate fatigue via maximal cardiopulmonary exercise testing and identify determinants of exercise intolerance in NAFLD. METHODS/STUDY POPULATION: In total, 14 subjects with histologically confirmed NAFLD were prospectively enrolled. Subjects with cirrhosis or those with known history of heart failure (systolic or diastolic) were excluded. Fatigue was quantified via the Duke Activity Status Index (DASI) questionnaire. A symptom-limited treadmill cardiopulmonary exercise test was performed in all subjects to measure exercise time (ET) and peak oxygen consumption (peak VO_2_). Doppler-echocardiography was performed to measure systolic and diastolic function. RESULTS/ANTICIPATED RESULTS: The DASI score and ET was significantly reduced in patients with NASH (n=10) when compared to those with SHS [40.2 (IQR=24.2–50.7) vs. 58.2 (IQR=50.7–58.2), *p*=0.04]; [9.1 (IQR=6.4–12.2) vs. 13.1 (IQR=12.5–13.1) min, *p*=0.02, respectively] reflecting moderate fatigue and impaired overall exercise capacity. The ET was directly linked to peak VO_2_ (*R*=+0.79, *p*<0.001), VO_2_ at anaerobic threshold (*R*=+0.73, *p*=0.003), and inversely to ventilatory efficiency index (*R*=−0.785, *p*=0.001) suggesting impaired cardiorespiratory fitness in those with reduced ET. ET was also linked to several parameters of diastolic dysfunction including left atrial volume index (*R*=−0.798, *p*<0.001), and the ratio of early transmitral pulse-wave Doppler flow velocity (E) to early mitral annulus tissue Doppler velocity E’ (E/E’) (*R*=−0.608, *p*=0.036), suggesting a role of diastolic dysfunction in patients with NAFLD with exercise intolerance. DISCUSSION/SIGNIFICANCE OF IMPACT: Cardiac abnormalities drive cardiorespiratory fitness and exercise intolerance in patients with NAFLD. These findings are exaggerated in patients with NASH suggesting a link between disease severity in NAFLD, exercise intolerance and diastolic dysfunction.

